# Accurately describing drug allergies and adverse drug reactions: The Australian Delphi Consensus on Drug Allergy Terminology

**DOI:** 10.1016/j.jacig.2025.100595

**Published:** 2025-10-31

**Authors:** Jillian Kehoe, Samantha Stiles, Sandra Vale, James Yun, Francis Thien, Jason Trubiano, Kiely Kim, William Smith, Michaela Lucas, National Allergy Council Drug Allergy Consortium, Alka Garg, Amanda Gwee, Andrew Carr, Annabelle Arnold, Bernadette Ricciardo, Connie Katelaris, Damian Chan, Elizabeth Healy, Frank Thien, James Yun, Jason Trubiano, Kiely Kim, Kirsten Perrett, Maia Brewerton, Michaela Lucas, Michelle Goh, Nick Cooling, Peter Cooke, Peter Goss, Peter Leman, Pravin Hissaria, Ray Mullins, Richard Scolaro, Sandra Salter, Sara Barnes, Sepehr Shakib, Shireen Sidhu, Suran Fernando, William Smith

**Affiliations:** a National Allergy Council, Sydney, Australia; b School of Population and Global Health, University of Western Australia, Perth, Australia; c Australasian Society of Clinical Immunology and Allergy (ASCIA), Sydney, Australia; d Department of Immunology, Prince of Wales Hospital, Sydney, Australia; e Faculty of Medicine and Health, The University of New South Wales, Sydney, Australia; f Department of Respiratory Medicine, Eastern Health, Box Hill Hospital, Melbourne, Australia; g Respiratory Medicine, Eastern Health, Monash University, Melbourne, Australia; h National Allergy Centre of Excellence, Melbourne, Australia; i Centre for Antibiotic Allergy and Research, Department of Infectious Diseases, Austin Health, Melbourne, Australia; j The Peter Doherty Institute for Infection and Immunology, Melbourne Medical School, University of Melbourne, Melbourne, Australia; k Allergy & Anaphylaxis Australia, Sydney, Australia; l Immunology Department, Royal Adelaide Hospital, Adelaide, Australia; m AllergySA, Adelaide, Australia; n Department of Immunology, Sir Charles Gardiner Hospital, Perth, Australia; o Department of Clinical Immunology, Perth Children’s Hospital, Perth, Australia; p Department of Immunology, PathWest Laboratory Medicine, Perth, Australia; q Medical School, Faculty of Health & Medical Sciences, University of Western Australia, Perth, Australia

**Keywords:** Consensus, Delphi, drug allergy, electronic health records, terminology

## Abstract

**Background:**

Standardizing drug allergy terminology is essential for consistent and accurate reporting of drug allergy information.

**Objective:**

This study aimed to define drug allergy–related terms for use in clinical care and allergy documentation using the Delphi process.

**Methods:**

The terminology list was developed by experts through a literature search, including relevant terms from the 10th and 11th revisions of the *International Statistical Classification of Diseases and Related Health Problems,* respectively. A multidisciplinary panel consisting of 30 experts with representation from pharmacy, pediatrics, clinical immunology and allergy, nursing, dermatology, infectious diseases, general practice, anesthesia, emergency medicine, and pharmacology was convened. An online Delphi process, consisting of 3 rounds of anonymous surveys and a final online discussion, was used to reach consensus, which was defined as group-level agreement of at least 75%. Data were collected between April 2023 and June 2024.

**Results:**

Over the course of the Delphi study, consensus definitions were reached for all 76 drug allergy terms. Of the consensus definitions, 3 (hypersensitivity, drug, and medication) required online group discussion to reach a consensus definition. Participation rates across the online surveys ranged from 90% and 60% in rounds 1A and 1B, respectively, 70% in round 2, and 66.7% in round 3.

**Conclusion:**

Consensus definitions for a broad range of commonly used drug allergy terms were established. This work addresses the urgent need for consistent drug allergy terminology to enhance the accuracy of recording of drug allergy in medical records, which is important for optimal patient care. This study provides the framework for future updates to ensure that the terminology evolves to meet the needs of diverse international health care settings, particularly as technology and health care continue to advance.

Drug allergies and adverse reactions are a growing problem worldwide, and they can result in life-threatening reactions that are often preventable, particularly for patients with known drug allergies.^1^^,^^2^ Although the true burden of drug allergy globally is unknown, it has been reported that 10% of people engaged with the health system declare that they have a drug allergy, accounting for 20% of emergency department visits for anaphylaxis.^2^ It is expected that this rate will increase with the emergence of new vaccines, antibiotics, and other novel drugs.^2^ Drug allergy comprises a complex set of clinical manifestations, and inaccurate or inconsistent reporting in medical records is common.^3^, ^4^, ^5^ Accurate documentation and transfer of allergy information across written and electronic health records (EHRs) is important for patient safety, as well as for enabling health professionals to coordinate care.^6^ The health professionals commonly involved in entering allergy information into health records include allergists, nonallergy specialists, doctors in training, nurses, general practitioners, pharmacists, and (in some instances) patients, thus resulting in a diverse knowledge base around recording allergy information.^7^ Standardizing drug allergy terminology is essential to maintain appropriate drug allergy management in health care settings, prevent allergen re-exposure, and ensure correct and accessible allergy information in patient health records. The widespread digital transformation of health records has enabled faster access to patient health information within and between health care settings.^6^ The accuracy of drug allergy information in EHRs is dependent on electronic health system useability and display (eg, pick lists and free text), accessibility and functionality of hardware, institutional guidelines and processes, clear and consistent terminology to appropriately describe allergic conditions, and health professional education and training (in both terminology and use of EHRs).^8^ National and international standards such as the Systematized Nomenclature of Medicine-Clinical Terms (SNOMED-CT) support consistency of terminology of electronic record documentation. Despite these standards, the recording of drug allergy information in EHRs is not consistent.^3^ Foreman et al highlighted the inappropriate use of terms by health professionals in a scenario-based study in South Australia, where it was found that 54.9% of obvious intolerances were documented in EHRs as an allergy and that free-text was used to describe reactions in more than a quarter of cases.^7^ Analysis of EHR in a large US health care system reported that severe cutaneous adverse reactions are documented as free text in most records, despite their high mortality rates.^9^ Promoting the use of standard terminologies is essential to improve how allergy information is recorded in EHRs, enabling better transfer of data between systems, accurate reporting, and triggering of appropriate alerts to health professionals, thereby improving clinical decision making.^6^^,^^9^ In response to the Parliamentary Inquiry to Allergies and Anaphylaxis, the Australian government and Australian Department of Health and Aging provided funding to form the National Allergy Council (NAC),^10^ which is a formal partnership between the Australasian Society of Clinical Immunology and Allergy (ASCIA) and Allergy & Anaphylaxis Australia (A&AA). The NAC was tasked with defining drug allergy terms for use in medical records. The NAC has also been working with the Australian Digital Health Agency and its collaborative partner organizations to standardize allergy documentation in EHRs. Thus, the aim of this study was to assemble multidisciplinary Australian and New Zealand experts to agree on drug allergy terminology for use in clinical care and allergy documentation by using the Delphi process. The intention was for the terminology to be used across primary, secondary, and tertiary health settings, thereby supporting multidisciplinary care through clear communication between health professionals, including specialists and nonspecialists, and patients. The intention was also to provide the nomenclature to be used in pick lists in electronic medical records and is therefore also directed at electronic medical record vendors and system designers. This work sets up a process for development and refinement of international relevant drug allergy terminologies, which will be useful to both health care professionals and researchers globally.

## Methods

### Pre-Delphi exercise

The initial list of drug allergy and adverse reaction terminology and associated definitions was identified on the basis of a literature search and included terms that had been agreed on earlier at a consensus meeting on drug allergy terminology in March 2020. Additional terms were collated from the 10th and 11th revisions of the *International Statistical Classification of Diseases and Related Health Problems* (ICD-10 and ICD-11, respectively). Proposed terms and definitions related to drug allergy were split into the following 4 subgroups: terminology, which described specific conditions (eg, severe cutaneous adverse reaction); symptoms (eg, benign rash); broad relevant medical terminology (eg, IgE); and operational or procedural terms (eg, drug challenge or delabeling). Definitions were kept concise and unambiguous, and they highlighted only major distinguishing characteristics. Additional information was provided alongside the definition for some of the terms, as determined by the pre-Delphi exercise.

### Expert panel

To obtain broad expertise, purposive sampling was used to recruit the Delphi panel. Panel members were recruited on the basis of the following criteria: drug allergy expertise, interest in the topic, and experience in managing patients with drug allergy. Experts were sent an e-mail invitation to be a Delphi panelist member and were provided with information about the study aims, time requirements, and importance of completing all rounds. Those who accepted the invitation and completed the Delphi rounds were included in the consortium.

Of the 34 invitees, 30 experts accepted the invitation; 2 experts declined the invitation, and 2 experts did not respond. Panelists represented the following health professional specialties with drug allergy experience: clinical immunology and allergy specialist (n = 12 [40.0%]); pediatrician (n = 3 [10.0%]); dermatologist (n = 3 [10.0%]); general practitioner (n = 2 [6.7%]); nurse (n = 2 [6.7%]); pharmacist (n = 2 [6.67%]); pharmacologist (n = 2 [6.7%]); anaesthetist (n = 2 [6.7%]); emergency physician (n = 1 [3.3%]); and infectious disease physician (n = 1 [3.3%]). There were 2 panelists from New Zealand (6.7%) and 28 from Australia, representing all jurisdictions except the Northern Territory: Victoria (n = 9 [30.0%]); South Australia (n = 6 [20.0%]); Western Australia (n = 5 [16.7%]); New South Wales (n = 4 [13.3%]); Queensland (n = 2 [6.7%]); Australian Capital Territory (n = 1 [3.3%]) and Tasmania (n = 1 [3.3%]).

### Delphi process

The Delphi process consisted of 3 rounds of anonymous online surveys, which were iteratively refined on the basis of panel responses, until consensus was reached. Online surveys were created and distributed using Qualtrics survey tools (Provo, Utah), through which the panelists voted on all terms and additional information. In each round, panelists were asked whether they agreed or disagreed with the proposed definition or had the option to indicate whether they were not qualified to answer the question. In those cases in which additional information was proposed, panelists were asked whether additional information was required and whether they agreed with the content of the additional information. If a panelist did not agree with the proposed definition and/or additional information, he or she was required to give a reason for disagreeing. Responses were collected and analyzed between survey rounds by the project team. Comments were used to revise definitions and additional information to take to the subsequent round. A final online meeting was convened to discuss any terms that did not reach consensus after the online Delphi survey process. Mentimeter (Stockholm, Sweden), a live online platform, was used to vote on terms, definitions and additional information in the final discussion round. The data were collected between April 2023 and June 2024.

Between June 2025 and July 2025, 2 additional rounds were conducted to obtain consensus on *Drug reaction with eosinophilia and systemic symptoms (DRESS)* and an updated definition for *skin tests (skin prick tests and intradermal tests)* to include the term *patch test*.

### Analysis

Consensus was defined as a level of agreement of at least 75%. Descriptive statistics were used to analyze closed-ended questions, in which case the percentage of agreement was calculated on the basis of the number of participants responding to each term per round. Missing values or responses from those respondents who indicated they did not consider themselves qualified to answer were treated as nonparticipation for that term. Open-ended questions captured panelist comments regarding suggested changes to the definitions. Responses to these questions were analyzed thematically by the 2 lead authors and were then synthesized and incorporated into revised definitions for subsequent rounds. The revised definitions were reviewed by the 2 immunology and allergy specialists leading the project. Data were recorded and analyzed using Microsoft Excel (Redmond, Wash).

## Results

**Fig 1:**
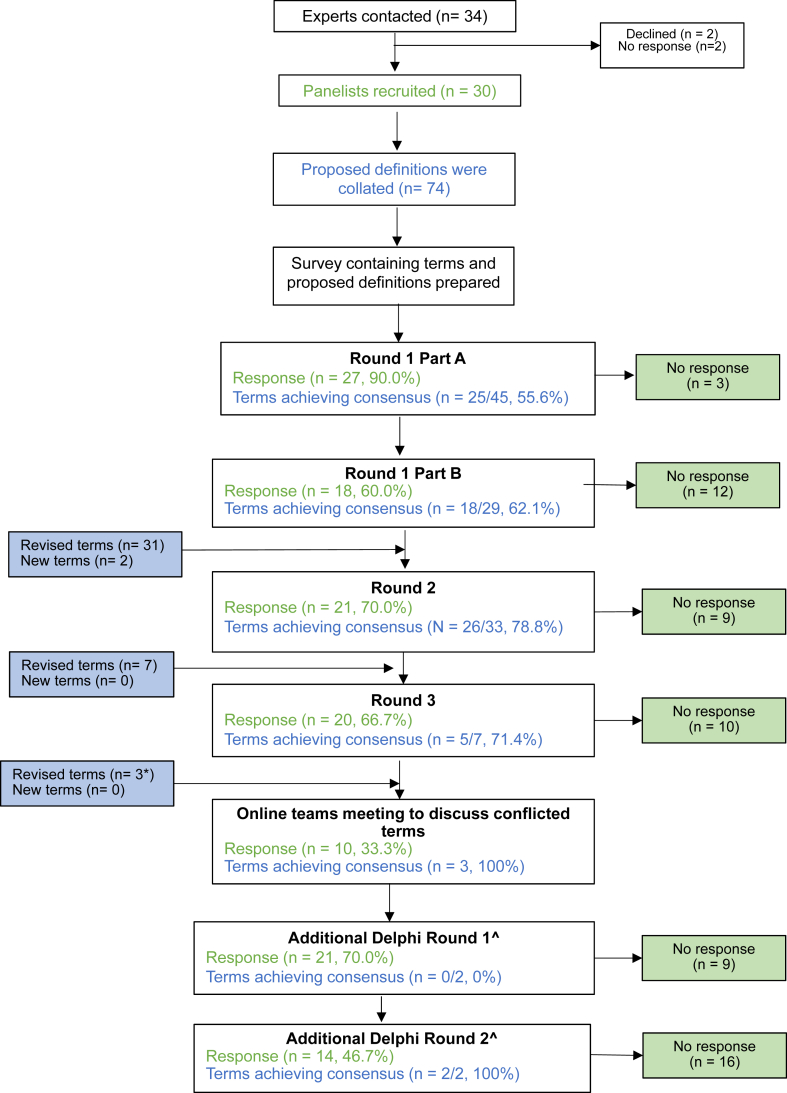
Flow diagram terms included and participants in each Delphi round.

The participation of panelists and the terms included and revised in each consecutive survey round are described in Fig 1. Because of the large number of terms, round 1 was split into 2 parts (part A and part B) to reduce the time to complete each survey and maximize participation rate. The participation rates in each round were as follows: 90.0% (27 of 30) in round 1 part A, 60.0% (18 of 30) in round 1 part B, 70.0% (21 of 30) in round 2, and 66.7% (20 of 30) in round 3. An additional online Delphi round, which consisted of 1 extra term and 1 updated term, was conducted after the discussion round. The participation rates for these additional rounds were 70.0% (21 of 30) in the additional round 1 and 46.7% (14 of 30) in the additional round 2. A total of 74 terms with definitions were presented to the Delphi panel initially, with 3 additional terms added during the Delphi process.

Over the course of the 3 anonymous online rounds, consensus was reached for 73 terms. For 5 of these terms, it was decided to include additional information. In all, 3 terms did not reach the predefined consensus threshold of 75% and were subsequently discussed online, with 10 of the 30 experts (33.3%) participating. This group included representation by 5 clinical immunology and allergy specialists, 2 pharmacologists, 1 anesthetist, 1 dermatologist, and 1 pediatrician. In all, there were 43 of 74 (58.1%), 26 of 33 (78.8%), and 5 of 7 (71.4%) terms that achieved consensus in round 1, round 2, and round 3, respectively. Consensus was reached for additional terms that went on to the subsequent Delphi (Fig 1).

The final consensus definitions are shown in Table I. The rates of agreement with the proposed definitions and additional information ranged from 76.0% to 96.3% in round 1, from 80.0% to 100.0% in round 2, and from 80.9% to 95.0% in round 3 (Table I). Panelists had the option of selecting the response “I’m not qualified to answer this question” if they did not consider themselves qualified. The terms that reached the higher proportion of “I’m not qualified” responses were *symmetric drug-related intertriginous and flexural exanthema* (42.9%), *drug-induced pemphigus* (33.3%), and *drug reaction with eosinophilia and systemic symptoms* (25.0%).

**Table I:** Drug allergy terms and definitions

Term	Consensus round	Consensus, %	Consensus definition
Condition terms			
Drug-induced aplastic anemia	2	100.0	Low levels of red blood cells, white blood cells, or platelets resulting from failure of bone marrow stem cells to generate new mature blood cells, caused by drug exposure through immunologic or nonimmunologic mechanisms
Drug-induced autoimmune hemolytic anemia	2	100.0	Hemolysis of red blood cells caused by drugs. This disease may present with pallor, fatigue, or shortness of breath
Erythema nodosum	2	100.0	A type of panniculitis, which is an inflammatory disorder affecting subcutaneous fat. It typically presents as painful red nodules on the anterior shins. Less commonly, it affects the thighs and forearms. It may be caused by drugs or viral infections, or it may be idiopathic
Erythroderma	2	100.0	Diffuse widespread erythema (redness) affecting all or most of the skin surface area (90% or more of skin surface area). Causes of erythroderma include drug reactions, inflammatory skin diseases (such as dermatitis and psoriasis), infections, or it may be idiopathic
Drug hypersensitivity	4	100.0	Adverse drug reaction which clinically resembles an allergic reaction. This can include both immunologic and nonimmunologic mechanisms
Cutaneous adverse drug reaction	1	96.3	Adverse drug reaction affecting the skin
Drug-induced vasculitis	2	95.0	Inflammation of blood vessels caused by taking a drug, resulting in changes in the walls of blood vessels. This most commonly affects the legs and may also affect other organs such as gastrointestinal tract or kidneys. Additional information: it arises after a variable period following drug commencement. May occur as part of a syndrome that includes fever, arthralgias, lymphadenopathy, elevated erythrocyte sedimentation rate, and rarely low complement levels
Drug intolerance	3	95.0	An undesirable pharmacologic effect that may occur at low or usual doses of the drug and is reproducible
Drug-induced nonautoimmune hemolytic anemia	3	93.0	Hemolysis of red blood cells caused by drugs with no involvement of autoimmune antibodies
Non–IgE-mediated drug allergy	1	92.6	Allergic drug reaction involving mechanisms other than type 1 (IgE-mediated) hypersensitivity; it may involve IgG antibodies (type 2 or 3) or T-cell (type 4) hypersensitivity mechanisms
Adverse drug reaction (ADR)	2	90.0	Harmful or negative effect of a drug, not including therapeutic failure, overdose or expected effect of medication
Drug-induced liver injury (DILI)	2	90.0	Drug hypersensitivity reaction causing hepatocellular, cholestatic, or mixed liver dysfunction that usually occurs at standard therapeutic doses
Acneiform reaction	1	88.9	Drug-induced acne-like rash characterized by follicular occlusion and inflammation
Non-steroidal anti-inflammatory drug (NSAID) hypersensitivity	1	88.9	Adverse reactions to aspirin/NSAID with clinical characteristics of allergy; caused by pharmacologic or immunologic mechanisms
Cross-reactivity (drug allergy)	2	86.0	An immediate (IgE-mediated) or nonimmediate (eg, T-cell) reaction specific to 2 or more structurally similar drugs or substances. May cause allergic reaction to a drug with no previous exposure
Severe cutaneous adverse reaction (SCAR)	2	86.0	A group of potentially life-threatening drug reactions affecting the skin and often mucosal surfaces as well. Systemic involvement and complications can be severe. SCARs include: - Stevens-Johnson syndrome/toxic epidermal necrolysis (SJS/TEN) - Drug reaction with eosinophilia and systemic symptoms (DRESS) - Acute generalised exanthematous pustulosis (AGEP) - Generalised bullous fixed drug eruptions (GBFDE)
Drug reaction with eosinophilia and systemic symptoms (DRESS)	2∗	83.3	Drug rash with eosinophilia and systemic symptoms (DRESS) syndrome is a severe T-cell–mediated drug reaction. DRESS typically occurs 2-8 weeks following exposure to the drug. It is typically accompanied by a variety of clinical manifestations, including fever, eosinophilia, lymphadenopathy, and organ involvement
Drug allergy	1	85.2	An immune-mediated adverse drug reaction. Additional information: typically, it includes immediate (IgE-mediated) or delayed (T-cell) mediated allergies
Fixed drug eruption (FDE)	1	85.2	Cutaneous allergic drug reaction characterized by well-demarcated erythematous macules that may develop into bullae and/or mucosal lesions, which recurs at the same site(s) with repeat exposure to the same (or a cross-reactive) drug
Nonallergic drug hypersensitivity	1	85.2	Adverse drug reaction that clinically resembles allergy but is not mediated by immunologic mechanisms
Drug-induced cytopenia	2	84.0	Low levels of red blood cells, white blood cells, platelets, and/or hematopoietic precursor cells in the marrow, caused by drug exposure through immune-mediated mechanisms
Aspirin-exacerbated respiratory disease (AERD)	1	81.5	Chronic asthma, usually associated with nasal polyposis, with acute exacerbation after taking aspirin or non-steroidal anti-inflammatory drug (NSAID)
Malignant hyperthermia due to anesthesia	1	81.5	A condition caused by hypermetabolism in response to certain anesthetic drugs, characterized by hyperthermia, tachycardia, tachypnea, increased carbon dioxide production, increased oxygen consumption, acidosis, muscle rigidity, and rhabdomyolysis
Multiple drug intolerance syndrome	1	81.5	Adverse drug reaction to 3 or more drugs that are neither structurally nor pharmacologically related, involving a nonallergic mechanism
Pseudoallergy	1	81.5	Adverse drug reaction clinically resembling allergy with a nonimmunologic mechanism
Stevens–Johnson syndrome (SJS) and Toxic epidermal necrolysis (TEN)	2	81.5	Severe mucocutaneous reaction most commonly caused by medications characterized by extensive necrosis and detachment of the epidermis. Mucous membranes are affected in over 90% of patients, usually at 2 or more distinct sites (ocular, oral, and genital). SJS and TEN are considered a disease continuum distinguished by severity, based on the percentage of body surface affected by blisters and erosions: - SJS is the less severe condition, in which skin detachment involves 10% of the body surface area- SJS/TEN overlap and describes patients with skin detachment involving 10%-30% of the body surface area. Additional information: a severe life-threatening skin condition and a medical emergency. Usually caused by drug allergy; however, occasionally caused by infection or malignancy
Drug anaphylaxis	2	81.0	Severe, systemic hypersensitivity reaction to a drug that occurs rapidly (usually within minutes to hours) after administration of the drug. Characterized by potentially life-threatening compromise in breathing and/or the circulation and is usually, although not always, associated with skin and mucosal changes. Commonly, but not always, IgE mediated
Drug tolerance	2	81.0	In the allergy context, the ability to take a drug without adverse reaction or symptoms. In the pharmacologic context, the progressively diminished effect of a drug over repeated or prolonged exposure
Drug-induced liver hypersensitivity disease	2	81.0	This an ICD-11 term that refers to an adverse drug reaction characterized by elevation of liver enzyme levels with symptoms ranging from mild to severe liver failure. Can occur concurrently with extrahepatic symptoms, including rash, fever, and multiorgan failure. It includes drug-induced liver injury
Co-reactivity (drug allergy)	2	80.0	Allergic reactivity to different drugs that is not due to cross-reactivity between the drugs. It may occur as part of multiple-drug hypersensitivity
Generalized bullous fixed drug eruption (GBFDE)	1	77.8	Cutaneous allergic reaction characterized by large, extensive, well-demarcated bullae with or without mucosal lesions, which recurs at the same site(s) with repeat exposure to the same (or cross-reactive) drug
Perioperative allergic reaction	1	77.8	Allergic reactions, including anaphylaxis, that occur during or shortly after surgical and interventional procedures
Lichenoid drug eruption	1	76.0	Cutaneous adverse drug reaction that has a delayed (by several weeks to 1 y) symptom onset and is characterized by a symmetric eruption of flat-topped, erythematous, or violaceous papules resembling lichen planus on the trunk and extremities
Medical terms			
Medication	4	100.0	A substance used to prevent, diagnose, or manage medical conditions. A medication may consist of active ingredients and other components (excipients)
Drug	4	100.0	A substance that has an effect when ingested or otherwise introduced into the body. In the context of allergy, the term *drug* is often used interchangeably with the term *medication*
Beta-lactam antibiotics	1	96.3	Antibiotics that contain a chemical structure called a beta-lactam ring: - Penicillin derivatives (penams) - Cephalosporins (cephems) - Monobactams - Carbapenems - Carbacephems
Pharmacovigilance (drug safety)	1	92.6	The pharmacologic science relating to collection, detection, assessment, monitoring, and prevention of adverse effects with pharmaceutical products
Tryptase	2	90.0	Enzyme that is abundant in mast cells and may be released into the blood when mast cells are activated by antigens binding to surface IgE. Tryptase release can occur in infections, autoimmune conditions, and immune-mediated systemic reactions such as anaphylaxis
Hapten	1	88.9	Small molecule bound to a larger molecule, forming an antigenic determinant; many drugs are too small to elicit an immune response, but they can do so when bound to a carrier molecule (usually a protein)
Human Leucocyte Antigen (HLA)	1	88.9	Glycoprotein receptor on the surface of human cells, responsible for presenting peptide antigens to the antigen-specific T-cell receptor, triggering an immune response. HLA molecules are encoded by polymorphic genes and vary between individuals
Pharmacologic interaction with immune receptors (P-i) concept	1	87.0	Drug hypersensitivity classification in which a drug binds noncovalently to an immune receptor, such as a T-cell receptor, which may lead to an immune response via interaction with a major histocompatibility complex molecule
Adverse drug event (ADE)	2	86.0	Harm experienced as a result of exposure to a drug
Type A adverse drug reaction	1	85.2	Predictable adverse reaction due to known pharmacologic properties
Adverse event following immunization (AEFI)	1	85.2	Adverse event occurring after immunization; no implication of causality
Type B adverse drug reaction	1	81.5	Unpredictable adverse reaction including drug allergy/hypersensitivity
Immunoglobulin E (IgE)	1	81.5	The subclass of antibody that is responsible for type 1 (immediate) allergic reactions. IgE can be measured in the blood and is bound to receptors on mast cells and basophils. Cross-linking of mast cell–bound IgE by a specific antigen (that can be a drug or drug hapten) results in degranulation of the mast cell, causing anaphylaxis
Complementary and alternative medicine	3	80.9	Medicinal products and practices that are not part of standard medical care. They are used in addition to traditional medical treatment (complementary) or instead of traditional medical treatment (alternative). These include a variety of substances purporting to have medicinal effect, but they may have less available scientific evidence about their safety and effectiveness compared to conventional treatments.
Cephalosporins	1	76.9	A large group of beta-lactam antibiotics. Some cephalosporins share side chains with penicillins, which leads to a risk of allergic cross-reactivity
Operational terms			
Drug desensitization	1	94.4	A method for inducing temporary tolerance in a patient with drug allergy by administration of incremental doses of the drug, starting from a dose below the threshold to cause a reaction and increasing to reach the therapeutic dose
Confirmed drug allergy	3	90.0	Drug allergy is considered certain or highly likely. The criterion standard is a drug challenge; however, in certain situations in which drug challenge may not be clinically safe or appropriate to perform, drug allergy confirmation may be based on: -A reliable history or observed reaction, and/or -A positive blood test result (eg, level of specific IgE, basophil activation) or skin test result in the presence of a consistent history
Drug challenge	1	88.9	Medically supervised test using protocols in which a drug is administered in a graded or single dose (parenteral or oral) while the patient is being monitored for adverse effects at each stage. Considered to be the criterion standard for drug allergy assessment. Additional information: drug challenge be done to confirm drug allergy or to delabel patients
Risk stratification	1	88.9	Determination of level of risk (likelihood and severity) that a patient is allergic to a drug
Skin tests (skin prick tests [SPT], intradermal tests [IDT] and patch test)	2†	79.0	Allergy tests used to identify allergens responsible for triggering allergic reactions, whereby the allergen is introduced in a prick through the epidermis (as in a skin prick test), injected into the dermis (as in an intradermal test), or topically applied to the skin (as in a patch test) to elicit a localized allergic response
Delabeling	1	83.3	The process of removing a drug allergy diagnosis from the patient’s medical record after assessment
Drug allergy not confirmed	1	77.8	Drug allergy is possible, but: - the patient’s history is uncertain or the reaction is said to have occurred in the distant past, or - skin testing or specific IgE blood testing yielded a negative result or has not yet been done, or - drug challenge not yet been done, or - a reaction was observed, but there are multiple potential culprits
Symptom terms			
Maculopapular rash	2	100.0	A generalized rash characterized by a combination of macules (flat, discoloured lesions) and papules (raised bumps)
Symmetrical drug related intertriginous and flexural exanthema (SDRIFE)	2	100.0	Systemic delayed hypersensitivity reaction with distinctive well-demarcated areas of erythema (redness) distributed symmetrically on the anogenital/buttock region and other skin fold areas (natal cleft, upper/inner thighs, axillae, or neck). It typically develops within hours to days of drug exposure and is self-resolving within days to weeks
Desquamate	2	95.0	Gradual, superficial shedding of the outer most layer of skin (epidermis)
Acute generalised exanthematous pustulosis (AGEP)	2	94.0	Allergic (T-cell–mediated) pustular drug eruption characterized by generalized pustular eruption, fever, and often neutrophilia. Usually arises within a few days (1-5 d) after drug initiation and becomes generalized (widespread). It is considered a severe cutaneous adverse reaction (SCAR), and its clinical features may overlap with those of other SCARs
Blister	2	90.0	Thin-walled sac on the skin containing fluid (clear or hemorrhagic). Blisters are classified as vesicles (typically less than 0.5 cm in diameter) or bulla (typically more than 0.5 cm in diameter)
Urticaria	3	89.0	Typically, itchy raised spots or patches of skin that may be red, pink, or pale in color and may disappear and reappear elsewhere. The spots usually resolve completely within 24 h, but they may be recurrent. Sometimes, but not always, due to an allergic reaction
Angioedema	1	88.9	Localized swelling (edema) of tissues beneath the skin or mucosa
Bulla	1	88.9	A large blister
Drug-induced fever	1	88.9	Pyrexia coinciding temporally with the administration of a drug, resolving after discontinuation. Usually suspected when other causes have been ruled out
Erythema multiforme	1	88.9	An acute, immune-mediated condition characterized by the appearance of distinctive, target-like lesions on the skin. These lesions are often accompanied by erosions or bullae involving the oral, genital, and/or ocular mucosae
Peel	1	88.9	Visible, exaggerated superficial shedding of the outermost layer of skin
Vesicle	1	88.9	Small blister less than 5 mm in diameter
Drug-induced pemphigus	2	86.0	Autoimmune blistering skin condition that can occasionally be induced or triggered by certain drugs, especially thiol drugs (eg, penicillamine, captopril). It may occur days, weeks, or months after the patient has started taking the drug. It causes painful blisters and erosions on the skin and mucous membranes
Drug-induced pruritus	2	85.0	Itching of the skin or mucous membranes triggered by a drug, with no visible rash
Drug-induced lupus erythematosus	2	84.0	Autoimmune response caused by certain drugs (eg, procainamide, hydralazine, and tumour necrosis factor inhibitors) that may result in a clinical syndrome with features of systemic lupus erythematosus (SLE), but without major organ-threatening complications. Symptoms may include fever, malaise, arthritis, arthralgia, myalgia, serositis, and rash
Drug-induced thrombocytopenic purpura	2	84.0	A skin condition caused by a low platelet count due to an immune-mediated (formation of antibodies) or non–immune-mediated (direct toxicity to megakaryocytes) drug reaction. Leads to accelerated platelet (or megakaryocyte) destruction. Can be mild to life-threatening
Contact dermatitis	1	83.3	Skin inflammation arising as a result of direct skin contact with an allergenic substance, including drugs. Additional information: a T-cell–mediated reaction. Usually has a delayed onset (12-24 h). May last for days to weeks after cessation of contact. Usually localized to the area of contact and may disseminate. Systemic contact dermatitis can occur from taking a drug to which the patient was initially sensitized through the skin. Allergic contact dermatitis is distinguished from irritant contact dermatitis (immediate reaction or chronic dermatitis caused by irritant exposure or injury to the skin)
Photosensitive drug reaction	1	83.3	Cutaneous reaction due to the interaction of ultraviolet radiation with drugs. Includes: - Phototoxic reaction (immediate) that appears as severe sunburn - Photoallergy immune-mediated delayed rash on sun-exposed areas
Pigmentary abnormality of skin due to drug	1	83.3	Disturbances of skin color resulting from an ingested or injected drug. These may result from a number of different mechanisms, including the color of the drug itself, disturbed melanization of the skin, or deposition of pigments by drug breakdown products
Benign rash	1	77.8	Transient mild or moderate rash that is not associated with blistering, mucosal involvement, or systemic features
Exanthem	1	77.8	A widespread rash with a range of causes, including toxins, drugs, infections, and autoimmune disease
Mucosal ulceration	1	77.8	A breach in the epithelial covering of mucosa (oral, conjunctival, genital, anal); it is often painful

*ADR,* Adverse drug reaction; *DRESS*, drug reaction with eosinophilia and systemic symptoms; *NSAID*, nonsteroidal anti-inflammatory drug; *SCAR*, Severe cutaneous adverse reaction; *SJS*, Stevens-Johnson syndrome; *TEN*, toxic epidermal necrolysis.

∗ This term was added after review of the article and sent to the Delphi panel for 2 rounds.

† Patch testing was added to this definition after review of the article and sent to the Delphi panel for 2 rounds.

The terms that reached the lowest consensus (between 75% and 80%) were *benign rash*, *cephalosporins*, *drug allergy not confirmed*, *exanthem*, *generalized bullous fixed drug eruption*, *lichenoid drug eruption*, *mucosal ulceration*, *perioperative allergic reaction*, and the updated definition of *skin test*; however, all of these terms reached consensus in round 1 (except for *skin test*, which reached consensus in round 2 of the additional Delphi process). The terms requiring the most refinement across 3 rounds were *complementary medicine*, *alternative medicine*, *confirmed drug allergy*, *drug intolerance*, *drug-induced nonautoimmune hemolytic anemia*, and *urticaria*, which finally reached consensus in round 3 (range 80%-95%). The terms *drug hypersensitivity*, *drug*, and *medication* required discussion during the final online meeting, as a result of which each reached a consensus of 100%. Because of the anonymity of the surveys, we cannot determine the precise number of panelists who reviewed each term across all rounds. However, we do know that the term *hypersensitivity* was reviewed 78 times, the term *medication* was reviewed 51 times, and the term *drug* was reviewed 31 times. The terms requiring additional information to support the concise definition were *contact dermatitis*, *drug allergy*, *drug challenge*, *drug-induced vasculitis*, *Stevens-Johnson syndrome*, and *toxic epidermal necrolysis* (Table I). The main points of disagreement across the rounds were as follows: (1) the terms were not definitive enough (too broad/generalized), (2) the definitions were too long or included irrelevant components, (3) there were factually incorrect components in the definition, (4) reference to “drug allergy” specifically was required, and (5) the language was too technical (it required simplification). Previously defined terms from local^11^ and global sources^12^, ^13^, ^14^, ^15^, ^16^, ^17^, ^18^, ^19^, ^20^, ^21^, ^22^, ^23^, ^24^ that informed the consensus definitions developed during this study are presented in Table E1 (available in the Online Repository at www.jaci-global.org) for comparison.

## Discussion

To our knowledge, this study is the first to standardize a large number of drug allergy terms with particular relevance to EHRs. Demoly et al (2014)^25^ previously conducted a review on behalf of the Joint Allergy Academies to gather health professionals’ attitudes toward allergy and hypersensitivity classification commonly used in routine allergy clinical practice.^26^ This review was primarily intended to guide development of the allergy and hypersensitivity chapter in the recent ICD-11 focusing on common conceptual misunderstandings between terms and the semantic framework for classifying terms in line with ICD coding hierarchies.^27^ Additionally, definitions for common drug allergy terms have been included in a number of guideline documents,^11^^,^^25^ including the most recent “Drug Allergy 2022: A Practice Parameter Update,” which was developed by the American Academy of Allergy, Asthma & Immunology and American College of Allergy, Asthma, and Immunology.^28^ Our study has incorporated international definitions related to drug allergy, including those listed in the current ICD-10 and updated ICD-11, thereby undertaking a unified approach to achieving expert consensus regarding a comprehensive list of 76 terms while still aligning with current international terminology. We have provided consensus definition on 45 additional drug allergy terms beyond the guidelines and terms included in the ICD-10 and ICD-11. Although this study was conducted in an Australian context, there is a need for consistent terminology globally—especially as health care systems become increasingly interconnected.

Importantly, this study involved input from a range of health professionals, with the goal of making these definitions applicable and used consistently across all health settings. This is essential, as common misconceptions regarding drug allergy–related terms still exist. Classification of allergic diseases is complex, as they involve a range of clinical presentations, mechanisms, and timing of onset. Inconsistent clinical definitions have led to inappropriate classification of drug allergy and varied terminologies used by different health professionals. As highlighted by the recent American Academy of Allergy, Asthma & Immunology position statement, the ability of EHRs to reliably capture accurate drug allergy information, which differentiates between strict avoidance for dangerous reactions or relative avoidance for mild intolerance is currently lacking.^29^ Misunderstandings between the concepts of allergy and hypersensitivity, which are often considered to be synonymous outside the allergy discipline, hamper proper classification of conditions in EHRs, and can lead to misinterpretation of perceived risks.^27^^,^^29^ In line with this, we found that the terms *hypersensitivity*, *drug*, and *medication* required the most discussion, going through 4 Delphi rounds. Clear definitions for the terms *drug allergy* and *drug hypersensitivity* as well as for *nonallergic drug hypersensitivity* provided in our terminology list are also vital when considering classification in the new ICD-11 chapter, which includes allergic and nonallergic hypersensitivity conditions.^27^ The inclusion of terms applicable to ICD-11, as well as the current ICD-10 was essential, as these provide the basis for allergy morbidity and mortality estimates globally. Although this terminology set was developed with EHRs in mind, it can be used for all health records, both written and electronic.

Currently, there is no standardization of allergy modules in EHRs across health care systems. The integration of up-to-date terms, based on concise consensus standardized definitions, with existing coding terminologies that are used globally, such as the ICD and the Systematized Nomenclature of Medicine-Clinical Terms, will improve data accuracy and strengthen interoperability within and between health care systems. This work is timely in the Australian context, as it coincides with the development of a national interoperability standard, and the integration of My Health Record, the national health information platform, with other clinical information systems across Australia. The NAC will continue to work with the Australian Digital Health Agency and their collaborative partner organizations to incorporate drug allergy terminology into clinical reference sets that will support these initiatives by integrating standardized drug allergy terminology across Australian health settings. Additionally, terminology will be incorporated into drug allergy training courses hosted by the ASCIA, which will be targeted toward both allergy and immunology specialists and nonallergy and immunology specialists. Training courses will be promoted through the ASCIA and NAC websites as well as through e-mail, conferences, and social media. The terminology and definitions will also be shared as health professional resources and supplement the best practice guidelines for the recording of, transfer of, and access to allergy information in EHRs, which that will be disseminated through the same communication platforms.

Beyond expanding terminology reference sets, another benefit of developing standardized definitions may be the ability to influence the future structure of allergy modules so that allergic conditions can be classified more appropriately.^7^ The Delphi process undertaken in this study represents a structured method to update and expand on drug allergy terms included in EHRs in the future, which is relevant in the Australian and global context. Given the diversity of health care systems, additional input from international Delphi panels could provide further refinement, ensuring that the terminology is applicable within different cultural and clinical contexts. Furthermore, the use of standardized drug allergy terminology, supported by health professional education, can improve access to accurate drug allergy data, creating opportunities for comparative drug allergy research across different countries.^25^ Standardized terminology can also underpin the development of future guidelines.

### Limitations

Although this study represents a significant step forward, acknowledging its limitations is important. Engagement levels varied across the 4 rounds of the Delphi process, potentially affecting the robustness of the results. For the fourth round, convening a meeting was difficult in view of the time differences across Australia; however, all panelists were given the opportunity to review and provide feedback even if they could not attend the meeting. Even with this limitation, only 3 definitions needed to be discussed in the fourth round. Notably, this study was carried out in Australia and New Zealand, where there is a strong connectivity between health care providers in the drug allergy field; however, further refinement of terms may be needed to ensure their applicability in other countries globally. For example, *in vitro* tests for drug allergy are currently not routinely available in Australia; hence, they were not included. Additionally, in Australia, the term *perioperative allergic reaction* is used more commonly than *perioperative hypersensitivity reaction*, highlighting the fact that the term *allergy* is commonly used instead of *hypersensitivity reaction*, including as an umbrella term for non–immune-mediated reactions. Future studies should include international panels to assess and adapt the terms for broader applicability. Although the definitions were kept as concise as possible, panelists thought that some of the more complex conditions required additional detail to ensure clarity for a broad range of health professionals, resulting in some definitions being longer. Finally, because the terminology is intended for use by health professionals, it may not be easily accessible or understandable to consumers. Longer definitions may be more challenging to standardize in EHRs.

### Conclusion

This research not only established standardized drug allergy terminology for Australia but also offers valuable information for health care professionals and researchers globally. This drug allergy terminology will be promoted by the ASCIA, as well as by clinical immunology and allergy specialists representing Australia and New Zealand. This Delphi panel has provided a framework for ongoing discussions and necessary refinements to ensure that the terminology evolves alongside clinical practices and modernized EHRs. This work could be a starting point for ongoing refinement of this list of 76 terms and definitions, continually assessing their ongoing relevance and effectiveness across diverse health care settings globally. The evolution of medical terminology owing to a variety of factors must be acknowledged, and this study sets the foundation for an ongoing unified approach through regular review and updates reflecting needs for different international health care settings, especially as technology and health care are advancing.

## Disclosure statement

The National Allergy Council has received funding from the Australian Government, Department of Health and Aged Care.

Disclosure of potential conflict of interest: The authors declare that they have no relevant conflicts of interest.

## Supplementary data

Consortium members

Table E1
