# Supplementary material for: Accurately describing drug allergies and adverse drug reactions: The Australian Delphi Consensus on Drug Allergy Terminology
Source: J Allergy Clin Immunol Glob. 2025 Oct 31;5(1):100595. doi: 10.1016/j.jacig.2025.100595 (PMC12743412; doi:10.1016/j.jacig.2025.100595)
Supplement: Consortium members [file mmc1.docx]

**National Allergy Council Drug Allergy Consortium**

## Alka Garg

Pharmacist

*SA Pharmacy, Women’s and Children’s Hospital, Adelaide, Australia*

**Amanda Gwee**

Pharmacologist

*Antimicrobials Group, Murdoch Children’s Research Institute, Melbourne, Victoria, Australia*

*Department of General Medicine and Infectious Diseases, Royal Children’s Hospital, Melbourne, Victoria, Australia*

*Department of Paediatrics, The University of Melbourne, Melbourne, Victoria, Australia*

*Amy Whittaker John Flynn Private Hospital, Queensland, Australia*

**Andrew Carr**

Clinical immunology/ allergy specialist

*Immunology and HIV Unit, St Vincent’s Hospital, Sydney, Australia*

*University of New South Wales, Sydney, Australia*

## Annabelle Arnold

Nurse

*Department of Immunology, Perth Children’s Hospital, Perth, Australia*

## Bernadette Ricciardo

Dermatologist

*Department of Dermatology, Fiona Stanley Hospital, Perth, Australia*

*Department of Dermatology, Perth Children’s Hospital, Perth, Australia*

*Wesfarmers Centre of Vaccines and Infectious Diseases, The Kids Research Institute Australia, Perth, Australia*

*School of Medicine, University of Western Australia, Perth, Australia*

## Connie Katelaris

Clinical immunology/ allergy specialist

*Campbelltown Public Hospital, Sydney NSW*

## Damian Chan

Clinical immunology/ allergy specialist

*The Women's and Children's Hospital, Adelaide, SA*

## Elizabeth Healy

Nurse

*Royal Children's Hospital, Melbourne, Victoria, Australia*

## Frank Thien

Clinical immunology/ allergy specialist

*Australasian Society of Clinical Immunology and Allergy, Sydney, NSW, Australia*

*Box Hill Hospital, Melbourne, Victoria, Australia*

*Monash University, Melbourne, Victoria, Australia*

## James Yun

Clinical immunology/ allergy specialist

*Nepean Hospital, Sydney, NSW, Australia*

## Jason Trubiano

Infectious diseases physician

*National Allergy Centre of Excellence, Melbourne, Victoria, Australia*

*Austin Health, Melbourne, Victoria, Australia*

*University of Melbourne, Melbourne, Victoria, Australia*

## Kiely Kim

Former Allergy Educator

*Formerly Allergy & Anaphylaxis Australia, Sydney, NSW, Australia*

## Kirsten Perrett

Paediatrician

*National Allergy Centre of Excellence, Melbourne, Victoria, Australia*

## Maia Brewerton

Clinical immunology/ allergy specialist

*Auckland City Hospital, Auckland, New Zealand*

## Michaela Lucas

Clinical immunology/ allergy specialist

*University of Western Australia, Perth, WA, Australia*

*National Allergy Council, Sydney, NSW, Australia*

*Australasian Society of Clinical Immunology and Allergy, Sydney, NSW, Australia*

*Sir Charles Gardiner Hospital, Perth, WA, Australia*

*Perth Children’s Hospital, Perth, WA, Australia*

*Pathwest Laboratory Medicine, Perth, WA, Australia*

## Michelle Goh

Dermatologist

*Department of Dermatology, St Vincent's Hospital Melbourne, Melbourne, Victoria, Australia*

*Department of Dermatology, Austin Health, Melbourne, Victoria, Australia*

*Department of Dermatology, Alfred Health, Melbourne, Victoria, Australia*

*Department of Surgical Oncology (Dermatology), Peter MacCallum Cancer Centre, Melbourne, Victoria, Australia*

*Department of Medicine, The University of Melbourne, Melbourne, Australia*

## Nick Cooling

General practitioner

*School of Medicine, University of Tasmania, Hobart, Australia*

## Peter Cooke

Anaesthetist

*Auckland City Hospital, Auckland, New Zealand*

## Peter Goss

Paediatrician

*Granada Medical Centre, Melbourne, Victoria, Australia*

## Peter Leman

Emergency Physician

*Australian Resuscitation Council, Melbourne, Victoria, Australia*

*University of Western Australia, Medical School, Internal Medicine, Perth, WA, Australia*

## Pravin Hissaria

Clinical immunology/ allergy specialist

*Department of Clinical Immunology and Allergy, Royal Adelaide Hospital, Adelaide, SA, Australia*

*SA Pathology, Adelaide, SA, Australia*

*University of Adelaide, Adelaide, SA, Australia*

## Ray Mullins

Clinical immunology/ allergy specialist

*Clinical Immunology and Allergy, John James Medical Centre, Canberra, Australia*

## Richard Scolaro

Anaesthetist

*Department of Anaesthesia, Sunshine Coast University Hospital, Sunshine Coast, Australia*

## Sandra Salter

Pharmacist

*University of Western Australia, Perth, WA, Australia*

## Sara Barnes

Clinical immunology/ allergy specialist

*Monash Lung Sleep Allergy and Immunology, Monash Health Department, Melbourne, Australia*

*Monash University, Melbourne, Australia*

## Sepehr Shakib

Pharmacologist

*School of Biomedicine, Faculty of Health Sciences, University of Adelaide, Adelaide, SA, Australia*

*Northern Adelaide Local Health Network, Adelaide, SA, Australia*

## Shireen Sidhu

Dermatologist

*Department of Dermatology, Royal Adelaide Hospital, Adelaide, SA, Australia*

## Suran Fernando

Clinical immunology/ allergy specialist

*Department of Clinical and Immunology and Allergy, Royal North Shore Hospital, Sydney, NSW, Australia.*

*Immunology Laboratory, Royal North Shore Hospital, New South Wales Health Pathology, Sydney, NSW, Australia*

*Northern Clinical School, Faculty of Medicine and Health, Sydney University, Sydney, NSW, Australia*

**William Smith**

Clinical immunology/ allergy specialist

*National Allergy Council, Sydney, NSW, Australia*

*Australasian Society of Clinical Immunology and Allergy, Sydney, NSW, Australia*

*Royal Adelaide Hospital, Adelaide, SA, Australia*

*AllergySA, Adelaide, SA, Australia*
