# Supplementary material for: Accurately describing drug allergies and adverse drug reactions: The Australian Delphi Consensus on Drug Allergy Terminology
Source: J Allergy Clin Immunol Glob. 2025 Oct 31;5(1):100595. doi: 10.1016/j.jacig.2025.100595 (PMC12743412; doi:10.1016/j.jacig.2025.100595)
Supplement: Table E1 [file mmc2.docx]

**Supplementary Material**

**Supplementary Table 1.** Previously defined drug allergy terms and definitions

| **Consensus term** | ***Current term** | **Current definition** | **Source type** |
| --- | --- | --- | --- |
| **CONDITION TERMS** |  |  |  |
| Aplastic anaemia | Aplastic anaemia | A disease caused by determinants arising after birth, in the antenatal period or genetically inherited factors leading to the inability of stem cells to generate new mature cells. This disease is characterised by low levels of red blood cells, white blood cells, and platelets. This disease may present with pallor, fatigue, dizziness, increased risk of infection or increased bruising or bleeding.^12^ | WHO ICD-11 code description |
| Drug-induced autoimmune haemolytic anaemia | Drug-induced autoimmune haemolytic anaemia | Acquired haemolytic anaemic, immune: condition characterised by antibodies that are directed against red blood cells in an autoimmune reaction leading to low levels of red blood cells. This condition may present with pallor, fatigue, shortness of breath. Confirmation is by identification of antibodies in a blood sample and positive Coombs test result.^12^ | WHO ICD-11 code description |
| Erythema nodosum | Erythema nodosum | A type of panniculitis, an inflammatory disorder affecting subcutaneous fat. It presents as tender red nodules on the anterior shins. Less commonly, they affect the thighs and forearms.^13^ | DermNet |
| Erythroderma | Erythroderma | Diffuse inflammatory erythemas: A group of disorders characterised by diffuse redness of the skin. They may be due to drugs, viral infections or circulating toxins but frequently a precise aetiology cannot be determined.^12^ | WHO ICD-11 code description |
| Drug hypersensitivity reactions (DHRs) | Drug hypersensitivity reactions (DHRs) | Drug hypersensitivity reactions are the adverse effects of pharmaceutical formulations (including active drugs and excipients) that clinically resemble allergy. It belongs to type B adverse drug reactions, which are defined by the World Health Organization as the dose-independent, unpredictable, noxious, and unintended response to a drug taken at a dose normally used in humans. It covers many different clinical phenotypes with variable onset and severity.^12^ | WHO ICD-11 code description |
| Cutaneous adverse drug reaction | Cutaneous symptoms | Cutaneous (skin) symptoms of a mild or moderate allergic reaction to a drug can include urticaria (hives) and angioedema. Rashes due to infections can be mistaken as an allergic reaction to a drug.^11^ | ASCIA terminology document |
| Drug-induced vasculitis | Drug-induced vasculitis | Drug-induced vasculitis is an inflammatory vasculopathy associated with drugs of almost every class and accounting for approximately 3% of the vasculitides. Although small vessel disease limited to the skin is the most common form, involvement of blood vessels in virtually every organ system may occur. It can present multiorgan involvement and the mortality is described in up to 10% of cases.^12^ | WHO ICD-11 code description |
|  |  | Drug-induced vasculitis typically presents as palpable purpura and/or petechiae, fever, urticaria, arthralgias, lymphadenopathy, elevated erythrocyte sedimentation rate, and low complement levels.^14^ | UpToDate topic review |
| Drug intolerance | Drug intolerance | Drug intolerance is an undesirable pharmacologic effect that may occur at low or usual doses of the drug. Immune mechanisms are not thought to be involved, and a scientific explanation has not yet been established.^11^ | ASCIA terminology document |
| Drug-induced non-autoimmune haemolytic anaemia | Drug-induced non-autoimmune haemolytic anaemia | N/A | No appropriate source |
| Non-IgE mediated drug allergy | Non-IgE mediated allergy | In non-IgE -mediated allergic reactions a patient may have similar clinical symptoms to IgE-mediated reactions, ranging from mild or moderate to severe allergic reactions, including anaphylaxis. These reactions do not involve IgE antibodies against the allergens.^11^ | ASCIA terminology document |
| Adverse drug reaction (ADR) | Adverse drug reaction (ADR) | A response to a medicine which is noxious and unintended, and which occurs at doses normally used in man.^15^ | WHO guidance document |
|  |  | Adverse drug reactions (ADRs) is a general term that includes all unintended effects of a drug except for therapeutic failures, intentional overdose, abuse of the drug or errors in administration. ADRs can be classified into two types:  -Type A reactions are predictable reactions and are based on known pharmacological properties of the drug.  -Type B reactions are unpredictable and include drug  ADRs include all unintended effects of drugs (medications) except for therapeutic failures, intentional overdose, abuse of the drug or errors in administration.^11^ | ASCIA terminology document |
| Drug-induced liver injury (DILI) | Drug-induced liver injury (DILI) | Drug-induced liver injury is a drug allergy and a leading cause of acute liver failure.  Antibiotics are the most common cause for DILI worldwide.  DILI can clinically present with hepatocellular, cholestatic or mixed liver dysfunction. | ASCIA terminology document |
| Acneiform reaction | Acneiform reaction | A group of related disorders characterised by follicular occlusion and inflammation.^12^ | WHO ICD-11 code description |
| Non-steroidal anti-inflammatory drug (NSAID) hypersensitivity | Non-steroidal anti-inflammatory drug (NSAID) allergy (intolerance) | Aspirin and other non-steroidal anti-inflammatory drugs (NSAIDs) can cause reactions in some people. Symptoms include flushing, itchy rashes, blocked/runny nose and sometimes severe asthma, usually within an hour of taking a tablet.  Aspirin allergy (more correctly referred to as aspirin intolerance) is more common in people with nasal polyps and asthma (AERD or Samter’s Triad).  Aspirin-induced respiratory disease (AERD) is characterised by aspirin or non-steroidal anti-inflammatory drug (NSAID) induced respiratory reactions in a patient with underlying asthma and/or rhinitis or sinusitis.^11^ | ASCIA terminology document |
| Cross-reactivity (drug allergy) | Cross-reactivity | Cross-reactivity is an important clinical problem and may result in an ADR in some patients who are allergic to structurally related drugs.  For example, some patients who are sensitised to penicillin may also have allergic reactions to cephalosporins, due to side chain cross-reactivity and rarely due to beta-lactam ring allergy.^11^ | ASCIA terminology document |
|  |  | Problem associated with the degree to which an antibody or antigen participates in cross reactions.^12^ | WHO ICD-11 code description |
| Severe cutaneous adverse reaction (SCAR) | Severe cutaneous adverse reaction (SCAR) | Non-immediate severe cutaneous adverse reactions (SCAR) are rashes that are associated with fever, flu-like and other systemic symptoms.  SCAR are potentially life-threatening, and require urgent specialist care.  SCAR often involve mucosal surfaces as well as the skin and include:  -Stevens-Johnson syndrome/toxic epidermal necrolysis (SJS/TEN)  -Drug reaction with eosinophilia and systemic symptoms (DRESS)  -Acute generalised exanthematous pustulosis (AGEP)  Generalised bullous fixed drug eruptions (GBFDE).^11^ | ASCIA terminology document |
| Drug allergy | Drug allergy | Allergic reactions to drugs are immune mediated responses to drugs in sensitised people. Reactions can be IgE mediated (immediate) or due to activation of cellular immunity (non-immediate). Symptoms range from mild-moderate to potentially life-threatening.^11^ | ASCIA terminology document |
| Fixed drug eruption (FDE) | Fixed drug eruption (FDE) | A fixed drug eruption (FDE) is an allergic reaction to a medication that usually recurs at the same site/s each time a particular drug is taken. The number of involved sites may increase over time.^11^ | ASCIA terminology document |
|  |  | The term fixed drug eruption describes the development of one or more annular or oval inflamed erythematous patches on the skin as a result of systemic exposure to a drug. The patches may develop into bullae. The inflamed patches normally resolve with post-inflammatory hyperpigmentation but typically recur at the same site(s), often with progressively more involved sites, following each re-exposure to the drug. In extreme cases (generalised bullous fixed drug eruption) the clinical picture may mimic toxic epidermal necrolysis. A large number of drugs have been implicated as triggers^12^ | WHO ICD-11 code description |
|  |  | Fixed drug eruption is characterized by erythematous and edematous plaques with a grayish center or frank bullae.  Lesions recur at exactly the same sites (typically lips and tongue, genitalia, face, and acralareas) with drug re-exposure  These site(s) develop post inflammatory pigmentation.  Fixed drug eruption can occur in response to sulfonamides, anticoagulants, and many other drugs.  The mechanism is unknown, although T cells residing in the skin produce interferon gamma.^14^ | UpToDate topic review |
| Non-allergic drug hypersensitivity | Non-allergic drug hypersensitivity | N/A | No appropriate source |
| Drug-induced cytopenia | Drug-induced cytopenia | Drug-induced cytopenia is a relatively common immune-mediated cytopenia and the target cells include erythrocytes, leukocytes, platelets and hematopoietic precursor cells in the marrow. The most frequent condition is the drug-induced immune thrombocytopenia and the most frequent implicated drugs are penicillin and structurally related drugs, quinine, quinidine, sulfonamide antibiotics, non-steroidal anti-inflammatory drugs and anticonvulsants^12^ | WHO ICD-11 code description |
| Aspirin-exacerbated respiratory disease (AERD) | Aspirin-exacerbated respiratory disease (AERD) | Aspirin-exacerbated respiratory disease (AERD), also known as Samter’s Triad, is a chronic medical condition that consists of three clinical features: asthma, sinus disease with recurrent nasal polyps, and sensitivity to aspirin and other non-steroidal anti-inflammatory drugs (NSAIDs) that inhibit an enzyme called cyclooxygenase-1. This sensitivity usually manifests as respiratory reactions that occur upon ingesting or inhaling an NSAID, though the exact cause of the reactions is not known.^16^ | AAAAI terminology document |
| Malignant hyperthermia due to anaesthesia | Malignant hyperthermia due to anaesthesia | A condition caused by hypermetabolism in response to certain anaesthetic drugs. This condition is characterised by hyperthermia, tachycardia, tachypnoea, increased carbon dioxide production, increased oxygen consumption, acidosis, muscle rigidity, and rhabdomyolysis. This condition may be associated with genetic mutation.^12^ | WHO ICD-11 code description |
| Multiple drug intolerance syndrome | Multiple drug intolerance syndrome | Multiple drug intolerance syndrome occurs in patients with intolerance to three or more neither structurally nor pharmacologically related drugs, with no confirmation of allergy after evaluation, and can possibly be  driven by patient anxiety.^11^ | ASCIA terminology document |
| Pseudoallergy | Pseudoallergic (anaphylactoid) reactions | Pseudoallergic (anaphylactoid) reactions are immediate systemic reactions that mimic anaphylaxis but are caused by non IgE-mediated reactions with the release of mediators from mast cells and basophils.  This term is rarely used and is incorrect as the anaphylaxis that results has the same characteristics as an IgE-mediated reaction.^11^ | ASCIA terminology document |
| Stevens–Johnson syndrome (SJS) and Toxic epidermal necrolysis (TEN) | Stevens–Johnson syndrome (SJS) | Stevens–Johnson syndrome (SJS) is a life-threatening skin condition, in which cell death causes the epidermis to separate from the dermis.  SJS is thought to be a hypersensitivity complex that affects the skin and the mucous membranes.  The most well-known causes of SJS are certain medications, but it can also be due to infections, or more rarely, cancers.^11^ | ASCIA terminology document |
|  |  | A spectrum of severe and life-threatening hypersensitivity disorders affecting skin and mucous membranes, most commonly precipitated by an idiosyncratic reaction to medication. Stevens-Johnson syndrome (SJS) always involves mucosal surfaces but the skin involvement is limited by definition to <10% body surface area (BSA). Toxic epidermal necrolysis (TEN) may sometimes spare mucous membranes but skin involvement is by definition >30% BSA. An intermediate form is recognised in which mucosal involvement is accompanied by skin involvement of 10-30% BSA (SJS-TEN overlap syndrome). All forms result in extensive sloughing and ulceration and carry a significant risk of fatal outcome.^12^ | WHO ICD-11 code description |
|  | Toxic epidermal necrolysis (TEN) | Toxic epidermal necrolysis (TEN) is a type of severe skin reaction. Symptoms include fever and flu-like symptoms, followed by blister and peel.^11^ | ASCIA terminology document |
|  |  | Toxic epidermal necrolysis (TEN) is an acute life-threatening skin disease with commonly quoted overall risk of mortality of between 25 and 30%, though the risk of fatal outcome is around 90% in the most severely affected patients (SCORTEN score >5). It is characterised by the rapid onset of extensive erythema, necrosis, and bullous detachment of the epidermis (> 30% body surface area). Commonly, the mucous membranes are also involved. Death may result from a combination of sepsis, fluid depletion and multi-organ failure. In two thirds of cases, TEN is triggered by a clearly identifiable drug allergy.^12^ | WHO ICD-11 code description |
| Drug anaphylaxis | Anaphylaxis | Anaphylaxis is a severe and immediate Immunoglobulin E (IgE) mediated allergic reaction.  Anaphylaxis can affect breathing and/or the heart and blood pressure.  Anaphylaxis is potentially life threatening and requires urgent medical attention.  Whist anaphylaxis is more likely when medication is given by intravenous (IV) or intramuscular injection (IMI), anaphylaxis to oral medications can also occur.  The most common causes of anaphylaxis are allergies to drugs (medications), foods and insect bites or stings.^11^ | ASCIA terminology document |
|  |  | Anaphylaxis is a severe, life-threatening systemic hypersensitivity reaction characterised by being rapid in onset with potentially life-threatening airway, breathing, or circulatory problems and is usually, although not always, associated with skin and mucosal changes.^12^ | WHO ICD-11 code description |
| Drug tolerance | Drug tolerance | Drug tolerance is defined as a state in which a patient will tolerate a drug without an adverse reaction.  Desensitisation can induce temporary, but not permanent drug tolerance.^11^ | ASCIA terminology document |
| Drug-induced liver hypersensitivity disease | Drug-induced liver hypersensitivity disease | Drug-induced hypersensitivity syndrome — DiHS, also called DRESS, is a severe drug hypersensitivity reaction involving rash, fever (38 to 40°C) and multiorgan failure.  The liver, kidneys, heart, and/or lungs are most often affected in DiHS/DRESS.^14^ | UpToDate topic review |
|  |  | Drug-induced liver hypersensitivity disease is a relatively rare condition, but can have serious consequences for the individual patient, public health, regulatory agencies and the pharmaceutical industry. It is characterised by elevation in serum alanine-aminotransferase (ALT), conjugated bilirubin, or combined bilirubin, ALT and alkaline phosphatase (AP) levels > 2 times the upper limit of normal (ULN) and the most frequent related drugs are halothane, tienilic acid, dihydralazine, diclofenac, and carbamazepine.^12^ | WHO ICD-11 code description |
| Co-reactivity (drug allergy) | Co-reactivity | Co-reactivity is rare and refers to if a patient reacts to structurally unrelated drugs. This has been described for T-cell mediated reactions, especially for drug reaction with eosinophilia and systemic symptoms (DRESS).^11^ | ASCIA terminology document |
| Generalised bullous fixed drug eruption (GBFDE) | Generalised bullous fixed drug eruption (GBFDE) | Generalised bullous fixed drug eruption (GBFDE) is a bullous type of fixed drug eruption (FDE), characterised by sharply defined bullae at the same site following administration of offending drug. Unlike FDE, GBFDE requires aggressive treatment.^11^ | ASCIA terminology document |
| Perioperative allergic reaction | Perioperative allergic reaction | Anesthesia represents a pharmacologically unique situation, during which patients are exposed to multiple foreign substances including anesthetics, analgesics, antibiotics, antiseptics, blood products, heparin, polypeptides, and intravascular volume expanders, which can produce immediate hypersensitivity reactions or anaphylaxis.^17^ | WAO article |
| Lichenoid drug eruption | Lichenoid drug eruption | Lichenoid drug eruption, also called drug-induced lichen planus, is an uncommon, cutaneous adverse effect of several drugs.  It is characterized by a symmetric eruption of flat-topped, erythematous or violaceous papules resembling lichen planus on the trunk and extremities.  The time interval between the initiation of the offending drug and the appearance of the cutaneous lesions varies from several weeks to a year or more and depends upon the class of drug, dose, host reaction, and concurrent medications.  Histologic examination reveals lichenoid interface dermatitis.^18^ | UpToDate topic review |
| **MEDICAL TERMS** | **MEDICAL TERMS** |  |  |
| Medication | Medication | A pharmaceutical product, used in or on the human body for the prevention, diagnosis or treatment of disease, or for the modification of physiological function.^15^ | WHO guidance document |
| Drug | Drug | A pharmaceutical product, used in or on the hu man body for the prevention, diagnosis or treatment of disease, or for the modification of physiological function.^15^ | WHO guidance document |
| Beta-lactam antibiotics | Beta-lactam antibiotics | Beta-lactam antibiotics are antibiotics that contain a chemical structure called a beta-lactam ring. They include:  - Penicillin derivatives (penams)  - Cephalosporins (cephems)  - Monobactams  - Carbapenems  - Carbacephems^11^ | ASCIA terminology document |
| Pharmacovigilance (drug safety) | Pharmacovigilance (drug safety) | Pharmacovigilance (also known as drug safety), is the pharmacological science relating to the collection, detection, assessment, monitoring and prevention of adverse effects with pharmaceutical products.^11^ | ASCIA terminology document |
| Tryptase | Tryptase | Tryptase is a proteinase that is abundant in human mast cells and basophils. Serum Tryptase is used as a marker for mast cell activation and rises in anaphylaxis particularly when caused by drugs and stings.^11^ | ASCIA terminology document |
| Hapten | Hapten | N/A | No appropriate source |
| Human Leucocyte Antigen (HLA) | Human Leucocyte Antigen (HLA) | Human leukocyte antigens (HLA) are encoded by a highly polymorphic set of genes and are expressed as cell surface receptors that present antigenic peptides to T cells in a restricted manner, which allows discrimination between self- and foreign antigens. HLA antigens are glycoproteins that reside on the surface of almost every cell in the body. The primary function of these antigens is to serve as recognition molecules in the initiation of an immune response. HLA antigens on specialized immune cells present peptides from foreign substances (e.g. viruses and bacteria) to effector cells of the immune system. Effector cells are then responsible for driving both the cellular and humoral arms of the response.^19^ | Encyclopedia of immunobiology |
| Pharmacologic interaction with immune receptors (P-i) concept | Pharmacologic interaction with immune receptors (P-i) concept | The p-i concept (pharmacologic interaction with immune receptors) is a recently proposed drug hypersensitivity classification in which a drug binds noncovalently to an immune receptor, such as a T cell receptor. This may lead to an immune response via interaction with a major histocompatibility complex molecule.^11^ | ASCIA terminology document |
| Adverse drug event (ADE) | Adverse drug event (ADE) | Any injuries resulting from medication use, including physical harm, mental harm, or loss of function]. ADEs, compared with medication errors, are a more direct measure of patient harm.^20^ | UpToDate topic review |
|  | Adverse event or experience | Any untoward medical occurrence that may present during treatment with a medicine but which does not necessarily have a causal relationship with this treatment.^15^ | WHO guidance document |
| Type A adverse drug reaction | Type A adverse drug reaction | Predictable reactions due to known drug properties.^11^ | ASCIA terminology document |
| Adverse event following immunisation (AEFI) | Adverse event following immunisation (AEFI) | An adverse event following immunization (AEFI) is defined as any untoward medical occurrence following immunization which does not necessarily have a causal relationship to the vaccine. The adverse event may be any unfavourable or unintended sign, abnormal laboratory finding, symptom or disease.^15^ | WHO guidance document |
| Type B adverse drug reaction | Type B adverse drug reaction | Unpredictable ADRs that include drug hypersensitivities reactions and drug intolerance.^11^ | ASCIA terminology document |
| Immunoglobulin E (IgE) | Immunoglobulin E (IgE) | Immunoglobulin E (IgE) is one of the five subclasses of antibody related to allergic reactions present in the blood, usually in very low concentrations and bound to the surface of cells such as mast cells.  Cross linking of bound IgE on the surface of mast cells leads to the release of allergic mediators, including histamine. This can trigger mild, moderate or severe (anaphylaxis) allergic reactions.  These reactions are known as IgE-mediated allergies.  Allergen specific IgE can be measured using skin testing or blood tests.^11^ | ASCIA terminology document |
| Complementary and alternative medicine | Complementary and alternative medicine allergy (unproven medicines) | While so-called complementary and alternative medicines (CAM), including herbal medicines, are often considered to be safe, ADRs including allergic reactions can occur.  It is therefore important to include questions about CAM in the patient’s clinical history.^11^ | ASCIA terminology document |
| Cephalosporins | Cephalosporins | Beta-lactam antibiotics are among the most commonly prescribed drugs, grouped together based on a shared structural feature, the beta-lactam ring. Cephalosporins are the largest group of beta-lactam antibiotics, cover a broad range of organisms, are generally well-tolerated, are easy to administer, and are thus frequently used.^21^ | UpToDate topic review |
| **OPERATIONAL TERMS** | **OPERATIONAL TERMS** |  |  |
| Drug desensitisation | Drug desensitisation | Drug desensitisation is a medically supervised treatment, using protocols for rapid administration of incremental does (parenteral or oral), of allergenic drugs.  The aim is to induce temporary immune drug tolerance, by which effector cells are rendered less reactive to allergic immune responses. | ASCIA terminology document |
| Confirmed drug allergy | Confirmed drug allergy | When drug allergy is indicated from the patient history (such as previous anaphylaxis to a drug) this is usually considered to be a confirmed drug allergy.  When drug allergy is uncertain, skin testing and/or a medically supervised test called a drug challenge can be conducted by allergy specialists in hospital clinics.  If, after specialist assessment, drug allergy is diagnosed, the drug must be avoided.  Documentation of a diagnosed drug allergy should be in My Health Record, GP and hospital records.^11^ | ASCIA terminology document |
| Drug challenge | Drug challenge (provocation test) | Drug challenges are medically supervised tests using protocols in which a drug is administered (parenteral or oral), while the patient is being monitored for adverse effects at each stage.  Drug challenges are also known as provocation tests and are considered to be the gold standard for drug allergy assessment.  Location of challenges depends on the pre-existing risk profile of the patient and should be performed in a suitably equipped hospital or clinic, which can manage severe reactions.  If a true drug allergy is diagnosed after a drug challenge, the drug must be avoided. Documentation of a diagnosed drug allergy should be in My Health Record, GP and hospital records. People with a diagnosed drug allergy should carry or wear medical identification jewellery or a card listing their drug allergies.^11^ | ASCIA terminology document |
| Risk stratification | Risk stratification | N/A | No appropriate source |
| Skin tests (skin prick tests [SPT] and intradermal tests [IDT]) | Skin tests (skin prick tests [SPT] and intradermal tests [IDT]) | A skin prick test (SPT) introduces a tiny amount of allergen into the skin, via a prick into the skin.  Intradermal skin testing (IDT) injects a defined amount of allergen into the dermal layer of the skin.  IDT is more sensitive than SPT as it delivers a larger amount of allergen but can carry a risk of anaphylaxis in highly sensitised individuals.  SPT and IDT elicit a localised allergic response in the form of a wheal (bump) and flare (redness) at the site of testing.  • IDT can also elicit a delayed inflammatory reaction in the skin in nonimmediate drug reactions.  • When drug allergy is uncertain, SPT or IDT or a medically supervised drug challenge can be conducted by clinical immunology/allergy specialists.^11^ | ASCIA terminology document |
| De-labelling | De-labelling | Drug allergy de-labelling is the process of removing a drug allergy diagnosis from the patient medical record, after assessment.  Assessment is achieved by allergy testing or subsequent safe exposure to the drug.  The patient should receive a written and dated confirmation if their drug allergy diagnosis ‘label’ is removed.  It is important that the updated drug allergy status is recorded in all medical records for each patient.^11^ | ASCIA terminology document |
| Drug allergy not confirmed | Drug allergy not confirmed | Drug allergy that has not been confirmed (from the patient history, skin testing or drug challenges), may not be a true drug allergy.^11^ | ASCIA terminology document |
| **SYMPTOM TERMS** | **SYMPTOM TERMS** |  |  |
| Maculopapular rash | Maculopapular rash | Maculopapular rash is a skin rash with a combination of macular (flat, red areas) and papules (raised bumps).^11^ | ASCIA terminology document |
| Symmetrical drug related intertriginous and flexural exanthema (SDRIFE) | Symmetrical drug related intertriginous and flexural exanthema (SDRIFE) | Symmetrical drug-related intertriginous and flexural exanthema (SDRIFE) is also known as Baboon syndrome because of its resemblance to the distinctive red buttocks displayed by female baboons,  SDRIFE is a systemic contact dermatitis with well-demarcated patches of erythema distributed symmetrically on the buttocks.  The cause of SDRIFE may be drug-related, induced by systemic administration of drugs, including hydroxyzine, penicillin and iodinated radio contrast media.^11^ | ASCIA terminology document |
| Desquamate | Epidermal Desquamate | Epidermal desquamation consists of the gradual invisible shedding of corneocytes from the outermost layers of the stratum corneum through controlled degradation of corneodesmosomes, the major cell–cell junctions in the stratum corneum.^22^ | Journal article |
| Acute generalised exanthematous pustulosis (AGEP) | Acute generalised exanthematous pustulosis (AGEP) | Acute generalised exanthematous pustulosis (AGEP), also known as pustular drug eruption and toxic pustuloderma, is a rare severe cutaneous adverse skin reaction (SCAR), that is mostly related to medication administration.  AGEP appears on average five days after a medication is started.^11^ | ASCIA terminology document |
|  |  | This uncommon reaction to systemic medication is characterised by fever (generally on the same day as the start of the rash) and multiple, small, non-follicular pustules that arise on a widespread inflammatory erythema centred on the upper trunk and body folds. It may be difficult to differentiate from acute generalised pustular psoriasis. It usually appears within 24 hours of drug exposure. Antibiotics are probably the commonest precipitants although many drugs have been implicated.^12^ | WHO ICD-11 code description |
| Blister | Blister | A blistering disease is a condition in which there are fluid-filled skin lesions.   - Vesicles are small blisters less than 5 mm in diameter. - A bulla is a larger blister. Note that the plural of bulla is bullae. - Blisters may break or the roof of the blister may become detached forming an erosion. Exudation of serous fluid forms crust.^13^ | DermNet |
| Urticaria | Urticaria | Urticaria (also known as hives or welts) is a raised, itchy rash that appears on the skin. Urticaria can be limited to one part of the body or spread across large areas of the body.^11^ | ASCIA terminology document |
|  |  | Urticaria, angioedema and other urticarial disorders: A heterogeneous group of disorders characterised by dermal and/or subcutaneous and submucosal oedema. The most common underlying mechanism is release of histamine from mast cells with consequent capillary dilatation and tissue oedema. This is responsible for the weals of spontaneous and most physical urticarias. A variety of other mechanisms are involved in other urticarial disorders.^12^ | WHO ICD-11 code description |
| Angioedema | Angioedema | Angioedema is a swelling (oedema) of the structures of the skin (dermis, subcutaneous tissue), mucosa and submucosal tissues.^11^ | ASCIA terminology document |
|  |  | Urticaria, angioedema and other urticarial disorders: A heterogeneous group of disorders characterised by dermal and/or subcutaneous and submucosal oedema. The most common underlying mechanism is release of histamine from mast cells with consequent capillary dilatation and tissue oedema. This is responsible for the weals of spontaneous and most physical urticarias. A variety of other mechanisms are involved in other urticarial disorders.^12^ | WHO ICD-11 code description |
| Bulla | Bulla | A bulla is defined as an air space in the lung measuring more than one centimetre in diameter in the distended state.^23^ | UpToDate topic review |
| Drug-induced fever | Drug-induced fever | An abnormal elevation of body temperature of unknown origin, often as a result of a pathologic process.^12^ | WHO ICD-11 code description |
| Erythema Multiforme | Erythema Multiforme | Erythema multiforme is a self‐limiting reactive inflammatory dermatosis triggered by cell‐mediated hypersensitivity, most commonly to drugs or infection, particularly Herpes simplex. It is characterised by an eruption of macules, papules, nodules, vesicles and/or bullae affecting preferentially the dorsal aspects of the hands and forearms. It may also involve oral and genital mucous membranes.^12^ | WHO ICD-11 code description |
| Peel | Peel | N/A | No appropriate source |
| Vesicle | Vesicle | N/A | No appropriate source |
| Drug-induced pemphigus | Pemphigus | A group of chronic autoimmune skin diseases characterised by blister formation on the skin and the mucous membranes. The exact causes of the disease are unknown but the disease is mediated by auto-antibodies to desmosome components. Three clinical forms have been characterised. Pemphigus vulgaris, pemphigus foliaceus and pemphigus vegetans. Other variants exist, namely intercellular IgA dermatosis and paraneoplastic pemphigus.^12^ | WHO ICD-11 code description |
| Drug-induced pruritus | Drug-induced pruritus | Pruritus attributable to drugs, in particular opioids  Pruritus: An intense itching sensation that produces the urge to rub or scratch the skin to obtain relief.^12^ | WHO ICD-11 code description |
| Drug-induced lupus erythematosus | Drug-induced lupus erythematosus | Acute cutaneous lupus erythematosus is the acute onset of rash, especially if widespread, and the frequent association with systemic symptoms and laboratory abnormalities may occasionally mimic DRESS.  The diagnosis of a cutaneous lupus erythematosus is confirmed by autoantibody testing and histologic examination of skin biopsy that demonstrates interface dermatitis with increased mucin and thickened basement membrane.  Direct immunofluorescence may show a continuous band of granular fluorescence at the dermoepidermal junction.^24^ | UpToDate topic review |
|  |  | Drug-induced lupus erythematosus is a syndrome in which positive antinuclear antibodies are associated with symptoms, such as fever, malaise, arthritis, intense arthralgia/myalgia, serositis, and/or rash. The syndrome appears during therapy with certain medications (e.g., procainamide, hydralazine, phenytoin) and tumour necrosis factor inhibitors. It occurs predominantly in Caucasians, has less female predilection than SLE, rarely involves kidneys or brain, is rarely associated with anti-dsDNA, is commonly associated with antibodies to histones, and usually resolves over several weeks after discontinuation of the offending medication.^12^ | WHO ICD-11 code description |
| Drug-induced thrombocytopenic purpura | Drug-induced thrombocytopenic purpura | Thrombocytopenic purpura attributable to drug toxicity (e.g. cytotoxic chemotherapeutic or immunosuppressive agents) or to an idiosyncratic drug-associated allergic thrombocytopenia (e.g. quinine, thiazides).^12^ | WHO ICD-11 code description |
| Contact dermatitis | Contact dermatitis | Contact dermatitis is a reaction to topically applied drugs, which is characterized by erythema and edema with vesicles or bullae that often rupture, leaving a crust. Subacute and chronic contact dermatitis are characterized by lichenification, erythema, and scaling.^14^ | UpToDate topic review |
| Photosensitive drug reaction | Photosensitivity due to drug | A photosensitive skin reaction to a medicament, most commonly a phototoxic reaction to a systemically administered drug, although photoallergy to drugs may rarely occur.^12^ | WHO ICD-11 code description |
| Pigmentary abnormality of skin due to drug | Pigmentary abnormality of skin due to drug | Disturbances of skin colour due to an ingested or injected drug. These may result from a number of different mechanisms including the colour of the drug itself, disturbed melanisation of the skin or deposition of pigments by drug breakdown products.^12^ | WHO ICD-11 code description |
| Benign Rash | Benign Rash | In the context of drug allergy, a benign rash is a transient morbilliform or maculopapular rash that may be mildly pruritic and is not associated with other symptoms. | ASCIA terminology document |
| Exanthem | Exanthematic drug eruption | Acute skin eruption typically resembling viral infections such as measles, rubella or scarlatina attributable to drug. Antibiotics are common causes.^12^ | WHO ICD-11 code description |
| Mucosal ulceration | Mucosal ulceration | N/A | No appropriate source |

WHO, World health organization; ICD-11, International classification of diseases (11th revision); ASCIA, Australasian Society of Clinical Immunology and Allergy; AAAAI, American Academy of Allergy, Asthma and Immunology; WAO, World Allergy Organization.

*Current term and definitions represent those that have been previously defined in local and global sources. These are provided to allow comparisons with consensus terms defined in this study.
